# Emerging Role of Genetic Alterations Affecting Exosome Biology in Neurodegenerative Diseases

**DOI:** 10.3390/ijms20174113

**Published:** 2019-08-23

**Authors:** Paola Riva, Cristina Battaglia, Marco Venturin

**Affiliations:** Dipartimento di Biotecnologie Mediche e Medicina Traslazionale, Università degli Studi di Milano, Via Fratelli Cervi 93, 20090 Segrate, Italy

**Keywords:** exosome biogenesis, exosome cargo, neurodegenerative diseases, genetic lesions, exosome pathway impairment

## Abstract

The abnormal deposition of proteins in brain tissue is a common feature of neurodegenerative diseases (NDs) often accompanied by the spread of mutated proteins, causing neuronal toxicity. Exosomes play a fundamental role on their releasing in extracellular space after endosomal pathway activation, allowing to remove protein aggregates by lysosomal degradation or their inclusion into multivesicular bodies (MVBs), besides promoting cellular cross-talk. The emerging evidence of pathogenic mutations associated to ND susceptibility, leading to impairment of exosome production and secretion, opens a new perspective on the mechanisms involved in neurodegeneration. Recent findings suggest to investigate the genetic mechanisms regulating the different exosome functions in central nervous system (CNS), to understand their role in the pathogenesis of NDs, addressing the identification of diagnostic and pharmacological targets. This review aims to summarize the mechanisms underlying exosome biogenesis, their molecular composition and functions in CNS, with a specific focus on the recent findings invoking a defective exosome biogenesis as a common biological feature of the major NDs, caused by genetic alterations. Further definition of the consequences of specific genetic mutations on exosome biogenesis and release will improve diagnostic and pharmacological studies in NDs.

## 1. Introduction

A common pathological feature of many neurodegenerative diseases (NDs) such as Alzheimer’s disease (AD), Parkinson’s disease (PD), Huntington’s disease (HD), amyotrophic lateral sclerosis (ALS) and prion diseases is the abnormal deposition of proteins in the brain, with a role in neurodegeneration. This condition is often associated with a spread of mutated or misfolded proteins via extracellular vesicles (EVs), causing neuronal toxicity. The role of the endosomal pathway is fundamental in processing and removing protein aggregates by lysosomal degradation or inclusion into multivesicular bodies (MVBs) and their release into the extracellular space as exosomes [1]. Depending on the donor cells and their contents, exosomes may display many different functions and very likely are important in intercellular interaction and maintenance of tissue homeostasis. In the central nervous system (CNS), exosomes and their cargo play a role in normal communication as well as nerve regeneration, synaptic function, plasticity and immune response [2]. The role of exosomes in health, ageing and disease is still under investigation, but recently they have been implicated in contributing to neurodegenerative disease spread and mental disorders [2,3,4].

In some pathological conditions, for example in AD [5], exosomes appear to be the most effective vehicle for removal the excess of intracellular amyloid β (Aβ) [6], leading to plaque formation [7]. Conversely, exosomes containing normal Aβ levels and neuroprotective factors have a function of cleaner for synaptotoxic Aβ species, promoting neuroprotection [8]. The impairment of this mechanism impacts on synapsis activity so that synaptic dysfunction is considered a hallmark in neurodegenerative disorders. Another important phenomenon mediated by exosome activity is the delivery of toxic forms of α-synuclein (α-syn) in the cerebro-spinal-fluid (CSF) of patients with PD and dementia with Lewy bodies [9]. Likewise, the functional impairment of exosomes in transferring proteins, mRNAs and miRNAs has been related to synaptopathies [10]. A similar exosomal function seems to mediate the transfer of pathogenic RNAs [11] or miRNAs with a neuroprotective role after ischemic brain injury. Exosomal transfer of miRNAs (i.e., miR-133b) from mesenchymal stromal cells (MSCs) to astrocytes and neurons mediates neurite remodeling and functional recovery after stroke [12], indicating that these vesicles may play a role in intercellular communication, being associated with different physiological and pathological functions [13].

Exosomes may acquire a potential role as biomarkers according to their protein and/or RNA content [14]. Abnormal miRNA profiles in exosomes from CSF and blood of patients with neurodegenerative disorders allow the identification of new biomarkers for diagnosis [15,16,17]. Furthermore, the potential capability of exosomes to deliver siRNAs provides a therapeutic potential tool for AD [18]. Proteins associated with AD, PD and prion diseases, Creutzfeldt–Jakob-disease (CJD) or bovine spongiform encephalopathy (BSE) can be selectively incorporated into intraluminal vesicles of MVBs and then released as exosomes into the extracellular environment. Because they can be isolated from circulating fluids such as serum, urine, and CSF, they provide a potential source of biomarkers for neurological disorders [2].

Another important issue is the use of exosomes to vehicle pharmacological molecules, improving cell-free therapies for neurological diseases. Interestingly, since MSCs are able to migrate into areas of brain injury, exerting therapeutic effects in different neurological disorders, MSC exosomes could be used as shuttle for pharmacological molecules [19,20] thanks to the exosome ability to pass through the blood–brain barrier without causing the immunological response.

The increasing evidence of exosome biology alteration in neurodegeneration suggests investigating genetic/epigenetic mechanisms regulating the different exosome functions in CNS. In this review, we will introduce the mechanisms of exosome biogenesis and their involvement in neuronal cross-talk. Finally, we will discuss the emerging role of exosome functional impairment caused by specific pathogenic mutations in neurodegenerative diseases.

## 2. Exosomes and Other Extracellular Vesicles

The production of EVs is a common cellular process conserved during evolution, being secreted by both prokaryotic and eukaryotic cellular organisms. In pluricellular organisms, EVs can be detected in several body fluids, including saliva, urine, blood, CSF, bile, breast milk, semen, amniotic fluid and ascites. These vesicles can be identified with different names mainly according to their origin (prostasomes, oncosomes), size (microvesicles (MVs), nanovesicles) and cellular/extracellular location (exosomes, ectosomes). Microparticle, MV and ectosome refer to vesicles of 150–1000 nm released by budding from the plasma membrane (PM) [21]. Small vesicles (30–100 nm) of endosomal origin secreted during reticulocyte differentiation after fusion of multivesicular endosomes or MVBs with the PM [22] are classified as exosomes. However, most cells can probably release both PM- and endosome-derived vesicles. The exosome release involves the formation of intraluminal vesicles (ILVs) in MVBs, transport of MVBs to the plasma membrane and fusion of MVBs with the plasma membrane. A critical point emerging in many studies is the correspondence between EVs and exosomes, but their origin cannot always be defined because of the extreme dynamicity of MVBs fusion process with the PM that is not so simple to detect. It is thus important to identify informative methods allowing to distinguish exosomes from MVs on the basis of size, structure, protein composition, their physiological relevance and mechanisms of biogenesis and secretion [23].

Exosomes can be rapidly purified from cell supernatants by means of serial filtration and immunoaffinity purification against specific surface markers. Fine exosome biochemical characterization is carried out by means of laser scatter tracking and other techniques such as mass spectrometry. A biochemical catalogue of exosome molecules is available in the database Exocarta (http://exocarta.org/) [24] and in a more comprehensive one, Vesiclepedia (http://www.microvesicles.org/, v4.1, 15 August 2018) [25]. The database Vesiclepedia includes data on proteins, nucleic acids and lipids, as well as on the purification procedures used, and it is continuously updated with the help of the scientific community. Exosome membrane, as well as cargo composition, reflects host cell identity (Table 1). The exosomal membrane contains cholesterol, sphingolipids, sphingomyelin, ceramides and its derivatives, glycerophospholipids. But unlike the plasma membrane, the exosomal membrane is relatively rigid at pH 7, conferring protection from degradation from the extracellular milieu [26]. Recent studies have demonstrated that the formation of lipid-based segregation (lipid-rafts) is implicated in sorting the cytoplasmic proteins into exosomes and in the budding of the endosomal membrane [27]. Proteome studies have revealed that exosomes contain a conserved set of proteins across species, and the proteins commonly identified in exosomes include vesicle trafficking proteins, cell surface receptors, antigen presentation molecules, proteins involved in membrane fusion process, cell surface endosome-associated proteins and adhesion proteins (integrin and tetraspanin superfamily). In addition, other intraluminal proteins include cytoskeletal elements (actin, tubulin), heat shock proteins and enzymes.

Exosomes content (cargo) is heterogeneous, being present lipids, proteins and nucleic acids. Their composition depends on both the cell type and cellular conditions [28], but how the cargo is sorted into the vesicles is poorly known. Lipids seem to play an important role in the sorting of specific proteins into exosomes. Sphingosine 1-phosphate (SP1) regulates cargo of CD63, CD81 and flotillin and their sorting into exosomes via inhibitory G protein (Gi)-coupled S1P receptors located on MVB membranes [29]. Exosomes carry proteins that undergo specific post-translational modifications (PTMs) and might be important for a long-distance communication such as cytokines, hormones, growth and transcription factors. Interestingly, exosomes do not invoke an immune response. A common feature of exosomal proteins is that all of them are ubiquitinated: This modification targets proteins of exosomal origin. Exosomes transfer functional miRNAs and mRNAs that do not always match the profile of parental cells [30]. Indeed, several miRNAs were found more highly represented in exosomes than in donor cells. Recent studies have indicated that the sumoylated form of heterogeneous nuclear ribonucleoproteins A2B1 (hnRNPA2/B1) is involved in the mechanism of selective miRNA export and the sequence of miRNAs drive their localization into exosomes [31]. These findings suggest that certain miRNAs have evolved to be packaged into exosomes to elicit a specific biological function. Furthermore, KRAS protein is involved in miRNA sorting into exosomes [32], promoting localization of the RISC component Argonaute 2 (Ago2) to MVBs [33]. Interestingly, hyperactivating KRAS mutations impair the localization of Ago2 to MVBs. decreasing Ago2 secretion in exosomes [33]. Exosomes can also functionally deliver retroviral RNA repeats and tRNA sequences to microenvironment, suggesting the role of exosomes in gene regulation processes and cellular crosstalk. In the CNS, the fine biological and biochemical characterization of exosomes and their cargo have elicited more attention for their role in the normal communication in the CNS as well as nerve regeneration, synaptic function, plasticity and immune response [2].

## 3. Mechanisms of Exosome Biogenesis

### 3.1. Formation of MVBs 

The formation of MVBs depends on the fate of endocytosis: Specific endocytic pathways may lead to the internalization of extracellular ligands or cellular components, for recycling to the PM, or degradation [34,35]. During maturation, early endosomes [36] accumulate ILVs in their lumen leading multivesicular endosomes or MVBs formation.

**Figure 1:**
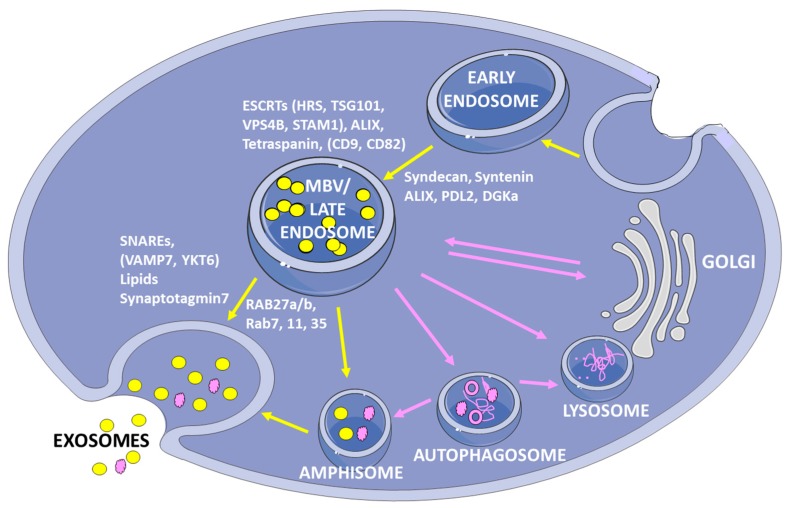
Schematic representation of exosome biogenesis and release. This pathway is traced by yellow arrows and includes exosome biogenesis starting from the invagination of endosome membrane, MBV transport and fusion with plasma membrane. The molecules mainly involved in specific steps of biogenesis are reported (see text for details). Microvesicles degradation rather than secretion is associated to cellular homeostasis, involving autophagosomes that can fuse alternatively with amphisomes (secretory pathway) or with lysosomes (degradation pathway). Homeostatic pathways are indicated by pink arrows.

The ILVs derive by inward budding of the early endosomal membrane, internalizing cytosol, lipids and proteins. MVBs fuse with lysosomes, causing their degradation. Conversely, MVBs, bearing the tetraspanin CD63, lysosomal-associated proteins LAMP1 and LAMP2, and other molecules generally present in late endosomes can also fuse with the PM, releasing their content into the extracellular compartment [37]. Different subpopulations of MVBs are present in cells at different living cycles or with different functions. Accordingly, some of these vesicles are destined towards the degradation pathway and others for exocytosis [23]. The biogenesis of exosomes is distinct on the basis of the involvement of ESCRT-dependent or ESCRT-independent mechanism, even if this classification cannot always be applied because the identification of the two molecular pathways is not so clear (Figure 1).

### 3.2. ESCRT Dependent Mechanism

Exosome biogenesis can be determined by endosomal sorting complex required for transport (ESCRT). The ESCRT mechanism involves about 30 proteins which assemble into four complexes (ESCRT-0, -I, -II, -III) involved in loading and budding of MVBs. Different ESCRT markers are present in exosomes from different cell types [38]. The depletion of HSR protein, an ESCRT-0 marker, reduces exosome release in HEK293 cells. Silencing of an ESCRT-I gene, *TSG101*, reduced exosome secretion, and the remaining secreted EVs reduced their cargo. ESCRT-II, ESCRT-III and VPS4 have the main role in the biogenesis of syndecan-, syntenin- and ALIX-containing exosomes [39]. Their implication is supported by the evidence that overexpression of syntenin increases ALIX-dependent exosome release, while the downregulation of ALIX, syntenin or syndecan impairs exosome release. The generation of exosomes through ESCRT-dependent mechanisms does not seem to determine the composition of their cargo. Colombo and colleagues reported that after a TSG101- or STAM1- knockdown in HeLa cells, a decreased amount of CD63 and MHC class II is detectable on recovered EVs, suggesting that TSG101 and STAM1 have a role in cargo loading [23]. Besides proteins, lipids are fundamental players in vesicular transport [39] since they are involved into membrane deformation, fission and fusion [40]. The inhibition of sphingomyelinase 2 (nSMase2), an enzyme involved in ceramide generation, leads to a reduction of exosomal release of proteolipid protein (PLP) from Oli-neu cells [27].

### 3.3. ESCRT-Independent Mechanism

The generation of EVs by mechanisms independent from ESCRT machinery involves membrane-associated proteins like tetraspanins besides other lipid raft-associated proteins, considered surface markers of exosomes. Their function can be inferred by the evidence that expression of tetraspanin 8 (Tspan8) causes modifications of transmembrane proteins (VCAM-1, α4 integrin) in the exosome content of a rat pancreatic adenocarcinoma cell line [41].

Tetraspanins play an important role in exosome biogenesis by ESCRT-independent mechanisms. CD81, an ubiquitary member of the tetraspanin family, is fundamental in forming molecular platforms for sorting specific cargoes, as well as in the formation of ILVs [42]. In CD81-deficient animals, exosomes are also found without CD81-interacting molecules, usually present within exosomes.

Furthermore, a small integral membrane protein of lysosomes and late endosomes (SIMPLE) was found to be secreted with exosomes. Fibroblasts from Charcot–Marie–Tooth disease patients expressing a SIMPLE mutated form are characterized by a lower secretion of CD63- and ALIX-containing exosomes, whereas flotillin amount remained the same [43]. SIMPLE has a role in the exosomal formation and contains a binding domain for TSG101 or NEDD4, interestingly the SIMPLE- Nedd4 interaction favors exosome secretion and the targeting of cytosolic proteins, among which PTEN tumor suppressor [44].

### 3.4. MVBs Transport

The fate of multivesicular bodies is the fusion with lysosomes, when their content has to be degraded, or with the plasma membrane for exosome release. A possible mechanism directing the MVBs towards the degradation pathway is the ISGylation, a posttranslational ubiquitin-like modification of MVBs, avoiding exosome secretion [45]. Transport of MVBs to the plasma membrane is dependent on their interaction with actin and microtubule cytoskeleton [46] and on membrane trafficking.

The Rab GTPases family is involved in membrane trafficking regulation, controlling vesicle budding, transport of vesicles along actin and tubulin, as well as membrane fusion [47]. Moreover, several Rab GTPases play an important role in exosome secretion. The involvement of Rab11 on exosomal secretion is supported by different findings [48]. Overexpression of a *RAB11* dominant-negative mutation in K562 cells [48] as well as its depletion in S2 cells of *Drosophila melanogaster* reduces exosome release [49]. The Rab11 control on exosome secretion seems to be cell-line-specific; in fact, Rab11 was not found to impair exosome release from HeLa cells [50]. Rab35 is involved in the release of exosome-associated proteolipid protein (PLP) from the oligodendroglial cell line Oli-neu, reducing MVBs docking at the plasma membrane [51]. In general, knockdown of Rab2b, Rab5a, Rab9a, Rab27a and Rab27b leads to a decreased exosome secretion [50]. Rab27a shows a reduced docking of MVBs to the plasma membrane in different cell lines [13]. The fusion of MVBs with the plasma membrane leading to the extracellular release of exosomes is mediated by a number of proteins involved in membrane fusion, including soluble N-ethylmaleimide-sensitive factor attachment protein receptors (SNAREs), Rabs and other Ras GTPases tethering factors [52].

### 3.5. Cellular Homeostasis

The destination of MVBs towards a degradation rather than a secretory pathway seems to be associated to cellular homeostasis. Both cellular stress and senescence increase exosome secretion [13]. This phenomenon is consistent with the involvement of exosomes in the protection of cells against intracellular stress [53,54]. In fact, the increasing in exosome release could contribute to eliminating waste products. Exosomes can be degraded by phagocytes or secreted for waste elimination purposes with the possibility of affecting neighboring cells, inducing pathological conditions, or might induce exosome release through a cellular communication about intracellular stress.

After triggering of autophagy, cytoplasmic cargo is trapped within double-membrane vesicles, the autophagosomes, which, if they fuse with MVBs, form amphisomes or differently they join lysosomes [55,56]. Induction of autophagy by starvation reduces exosome release, probably because the fusion between MVBs and autophagosomes increases, since directing MVBs to a degradative pathway [13]. Moreover, the autophagic machinery is also involved in secretory autophagy, caused by a specific secretion process releasing cytoplasmic substrates in the extracellular environment [57]. This process is induced when lysosomal dysfunction occurs in the cell. This is considered an alternative way as exosomes release waste products. Secretory autophagy might play a role in some NDs associated with dysfunctional autophagy and aggregation of proteins [58]. In the presence of lysosomal defect lysosomes, overload material and both lysosomal transport and secretion is impaired. In this condition, MVBs or amphisomes could be a way to rescue the cell. All these pathways are probably connected and finely regulated by complex mechanisms [13].

## 4. Exosome Function in CNS

**Figure 2:**
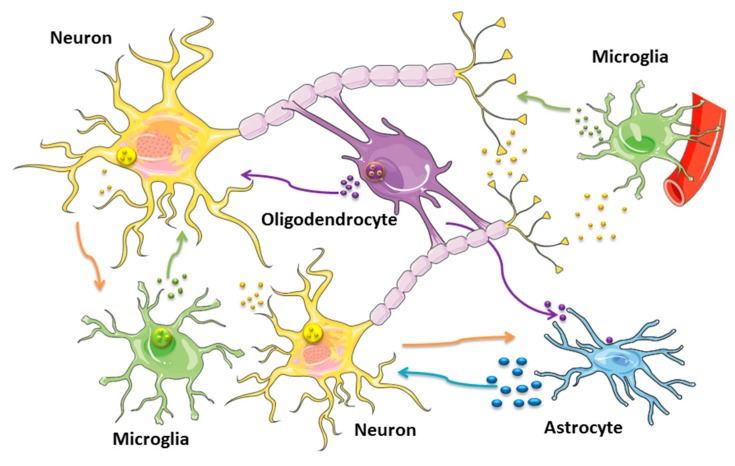
Exosomes in intercellular cross-talk involving CNS cells. The schematic representation illustrates the release of exosomes from major neural cells such as neurons, astrocytes, microglia and oligodendrocytes. NDEs (yellow particles and arrows) can be released from both the somato-dendritic compartments of neurons and from the presynaptic regions and can modulate activity of microglia, astrocytes and oligodendrocytes. ODEs (violet particles and arrows) can transfer myelin proteins and trophic factors to other neurons and astrocytes, providing metabolic supports. ADEs (blue particles and arrows) containing heat shock proteins and synapsin I can be taken up by neurons promoting their survival and development. MDEs (green particles and arrows) are involved in the neuroimmune communication, influencing neurite outgrowth, modulating neuronal activity and firing. See Table 1 and text for complete details.

Current evidence for exosome signaling in the brain points toward their role in transcriptional regulation, neurogenesis, plasticity and neuro immunomodulation [3]. The development and maintenance of neuronal circuits in the CNS require a complex series of events involving coordinated short- and long-distance communication between numerous cell types. A variety of CNS cell types including neurons and glial cells (microglia, astrocytes and oligodendrocytes) are able to release EVs (Figure 2). While neurons are highly specialized cells that are in charge to rapidly receive and transmit impulses to and from different parts of the body through chemically-mediated electric signals, glial cells are active, providing nutrients to the neurons, form myelin and, by insulating axons, assist in the propagation of the electric communication. Recent work has revealed that neurons and glial cells are almost equal in the human cortex, but the distribution can vary in different brain areas. Neuronal cells release different types of EVs, such as exosomes that could have an impact on synaptic activity, in neurogenesis, and in the overall regulation of neurological activities. While CNS derived exosomes display common exosome markers (ALIX, TSG101, tetraspanins, Rab GTPase proteins), their cargos are characterized by specific molecules (Table 1). The release of exosomes by donor CNS cells might be regulated by synaptic glutamatergic activity and calcium influx [59], and they can be found in peripheral circulation delivering molecular information within and across organs. EV release by neurons and glial cells may contribute to the spreading of toxic aggregates, as well as influence the aggregation process and the clearance of the aggregates. This feature has sparked an interest to explore the role of exosomes as Trojan horses of neurodegeneration: Genetic or environmental factors could modify exosome sorting and/or composition, affecting the fate of the aging neurons [60].

### 4.1. Neurons-Derived Exosomes (NDEs)

NDEs mediate a generalized neuron-glia crosstalk regulating neuronal regeneration and synaptic functions in development and adult life [3,60]. Their release is stimulated by potassium-induced depolarization, and they contain a wide variety of molecules playing important roles for neuronal function (Table 1). Exosomes released by cortical neurons are characterized by having adhesion molecules such as the L1 cell adhesion molecules or neural cell adhesion molecules (NCAM1 and NCAM2), AMPA receptors GluR2/3 subunits of glutamate receptors and microtubule-associated protein-1 (MAP1B). Beside the classical secretory pathway, Cystatin C (CysC) that is an endogenous cysteine protease inhibitor expressed in various tissues, was also found secreted by mouse primary neurons in association with exosomes [61] and seems to display a neuroprotective role in NDs such as AD [62]. Moreover, a trans-synaptic vesicular transport of Wnt signals by the trafficking protein Evi/Wntless has been reported in *Drosophila* larvae [63]. Wnt signaling orchestrates a myriad of development processes displaying an essential role in the neurodevelopment and might be involved in major NDs such as AD. The shuttling of synaptotagmin 4 (Syt4) was found from the presynaptic motor neuron to post-synaptic target cell (i.e., muscle) by means of NDEs [64]. Moreover, mRNAs could be packaged in NDEs in association with the activity-regulated-cytoskeleton-associated protein (Arc) that acts as a key regulator of synaptic plasticity [65]. Arc regulates synaptic plasticity by promoting endocytosis of AMPA receptors (AMPARs) in response to synaptic activity. Communication between neurons and microglia is bidirectional, and exosomes might be novel fine-tuning actors of neuroimmune communication pathways (Figure 2). Exosomal miRNAs from cortical neurons might be taken up by different CNS cells such as astrocytes (miR-124a), thus regulating extracellular glutamate levels and modulating synaptic activation (Figure 2); microglia cells can be other recipient cells involved in facilitating synapse elimination by up-regulation of complement factors.

### 4.2. Oligodendrocytes-Derived Exosome (ODEs)

ODEs are produced upon stimulation with the neurotransmitter glutamate. Glutamate is able to induce Ca^2+^ influx in oligodendrocytes, followed by activation of the small GTPase Rab35, leading the exosome release [66]. ODEs are enriched in myelin proteolipid protein 2′,3′-cyclin nucleotide 3′-phosphodiesterase, myelin-associated glycoprotein and myelin oligodendroglial glycoprotein. Remarkably, ODEs carry a range of enzymes including the NAD-dependent deacetylase sirtuin-2 (SIRT2), oxidative stress alleviating peroxiredoxins (PRDX-1 and -2) and dihydropyrimidinase-related proteins (DPYL-2 and -3), superoxide dismutase (SOD 1), catalase and glycolytic enzymes. Their neuronal uptake seems to play a role in protecting neurons during oxygen and glucose deprivation [67]. ODEs have been shown to participate in the myelin debris clearance mediated by microglia.

### 4.3. Microglia-Derived Exosomes (MDEs)

MDEs play a role in neuroimmune cross talks by targeting cells such as neurons and other glial cells. Proteomic profiling of MDEs identified a number of enzymes, chaperones, tetraspanins and membrane receptors [68]. MDEs can modulate neuronal activity also via enhanced sphingolipid metabolism. MDEs contain common exosomal proteins as well as a set of proteins previously reported for B cell- and dendritic cell-derived exosomes. The expression of CD13 and monocarboxylate transporter (MCT1) is unique to microglia. CD13 is an aminopeptidase involved in cleaving leucine- and methionine-enkephalin peptides. Thrombospondin 1 and -4 are contained in MDEs and play a role in suppressing neuronal apoptosis and promoting neurite outgrowth and synaptogenesis. Moreover, MVs carrying the proinflammatory cytokine IL-1β shed from the plasma membrane of microglial cells and astrocytes in response to ATP stimulation and activation of acid sphingomyelinase. Inflammatory MDEs transfer their miRNA cargo (miR-146a-5p) to neurons, determining the loss of excitatory synapses, suggesting a role during brain inflammation probably by silencing key synaptic genes [69]. The release of α-syn from MDEs has been recently demonstrated as a mechanism that might facilitate protein aggregation in PD [70].

### 4.4. Astrocytes-Derived Exosomes (ADEs)

ADEs have both homeostatic and pathogenic functions. ADEs release may be generated in response to a sustained increase of intracellular [Ca^2+^] or by mechanical stimulation [71]. Their cargos are enriched in heat shock proteins (HSP70), facilitating neuronal survival. Synapsin-1 is a neuronal phosphoprotein that coats synaptic vesicles, binds to the cytoskeleton, and is believed to function in the regulation of neurotransmitter release. Intriguingly, synapsin 1 is first released from the exosomal cytosolic compartment under these conditions, before it acts on neurons in its soluble form [72]. Many different factors are carried by ADEs that might regulate the activity of neighboring cells including growth factors such as FGF2, VEGF and angiogenetic inhibitor endostatin. ADEs contain miRNAs that are utilized in astrocyte-neuro crosstalk: For instance, miR-24b released via exosomes from astrocytes is taken up by neurons that in turn induces the downregulation of the expression of PDGF receptors [73]. ADEs are released upon oxidative stress and heat stress as well as in pathological conditions. The release of PAR4 -and ceramide–enriched exosomes is increased in amyloid plaques in a model of AD that in turn induces neuronal apoptosis, suggesting a possible contribution to the onset of the disease [74].

In the context of NDs, it appears that pathogenic proteins such as Aβ, prion protein (PrP), α-syn, Tau, superoxide dismutase-1 (SOD1) are released from cells in association with exosomes by different CNS cells (Table 1). These proteins have in common the property to form aggregates (amyloids, prionoids) that escape the normal cellular degradation machinery. Noteworthy since miRNAs regulate the level of proteins by regulating the levels of the corresponding transcripts, the alteration of their release via exosomes (esRNA) in turn might trigger alteration in exosomal protein content. However, very little is known of the potential role of esRNA in the pathogenesis and diagnosis of neurodegenerative disorders [75].

## 5. Genetic Defects in ND Genes Affecting Exosome Pathway

There is emerging evidence that mutations in genes known to be involved in the pathogenesis of- or susceptibility to NDs can lead to impairment of exosome production and secretion (Table 2), opening a new perspective on the mechanisms whereby genetic defects of these genes turn on the neurodegenerative process. In the following paragraphs, we will summarize the recent findings on the most common types of NDs, which indicate that compromised exosomal compartment might be a previously uncovered common thread of these genetic conditions.

### 5.1. Alzheimer’s Disease (AD)

AD is the leading cause of dementia worldwide, accounting for 60%–80% of all dementia cases [76]. Mutations in the genes encoding Aβ precursor protein (*APP*) and presenilins 1 and 2 (*PSEN1* and *PSEN2*) are the main cause of the familial form of the disease, which is a genetic condition inherited in an autosomal dominant manner with early-onset [77]. On the other hand, several risk loci have been identified so far for sporadic AD, which is a late-onset polygenic condition [78,79].

The main pathological hallmarks of AD are extracellular senile plaques, made by fibrillary β-amyloid (Aβ), and neurofibrillary tangles (NFTs), composed of hyperphosphorylated Tau [80]. Emerging evidence demonstrates that exosomes may participate in the AD process as well by contributing to the release of Aβ and Tau into the extracellular space and to their spread through the brain. Moreover, overexpression of human Tau in neuroblastoma cells has been shown to recruit proteins relevant to neurodegeneration into the exosomal secretion pathway [81]. On the other hand, several studies have provided evidence that neuronal exosomes may also exert a protective role in AD neurodegeneration by capturing Aβ and promoting its uptake by microglia, thus decreasing Aβ and amyloid deposition [82].

Among the different genetic risk factors of late-onset AD so far identified, allele 4 of *APOE* is widely recognized as the strongest one. The *APOE* gene encodes three major alleles (ε2, ε3, ε4). *APOEε4* heterozygous carriers have three times increased risk of AD, while the homozygous carriers a 12-fold increased risk.

Conversely, *APOEε2* is associated with decreased risk for AD and later age at onset. APOE plays several important roles in CNS, such as lipids and cholesterol transport, synaptic plasticity, synaptogenesis and inflammation. Several studies on humans and animal models demonstrate that APOE may influence the synthesis, clearance and aggregation of Aβ. APOEε4 shows less efficiency in the clearance of Aβ, resulting in excessive deposition of Aβ. APOE also indirectly affects the metabolism of Aβ by receptor-mediated interaction, such as a low-density lipoprotein receptor. In APP transgenic models, different APOE isoforms showed different effects on Aβ deposition. Similarly, *APOEε4* carriers show accelerated Aβ deposition compared with noncarriers in both neuropathological and neuroimaging studies [78].

Peng and collaborators have recently compared brain exosome levels in heterozygous or homozygous *APOEε4* carriers with those in people homozygous for *APOEε3* [83]. Frontal cortices of *APOEε4* carriers (either *APOEε4* homozygous or *APOEε3*/*Eε4* heterozygous) contained significantly lower levels of exosomes than those of non-carriers (APOEε3 homozygous). These data were further confirmed by the reduced levels of exosome markers ALIX and TSG101 found by western blot analysis in *APOEε4* compared with *APOEε3* carriers. Similar results were obtained in mice expressing humanized *APOE* alleles, indicating that the observed effects on exosome levels are solely dependent on *APOE* genotype. In mice, the levels of exosomes and exosome markers ALIX and TSG101 were significantly lower in the brains of 12-month-old and 18-month-old *APOEε4* compared with age-matched *APOEε3* mice, while they did not differ with genotype at six months of age, indicating an age-dependent effect of *APOEε4*. Altogether, these findings demonstrate that APOEε4 expression leads to lower exosome levels in the brains of both humans and APOE mice, with the mice data further suggesting that the effect is aging-dependent. Furthermore, regulators of intracellular exosome formation were downregulated in mice that carried APOEε4 at both mRNA and protein level, demonstrating that the decrease in exosome levels is caused by the impairment of exosome biogenesis. In addition, extracellular vesicle membranes in *APOEε4* carriers contained higher levels of cholesterol and ceramide than those in non-carriers, demonstrating that APOE4 compromises brain exosome production in a manner that may be linked to APOEε4-mediated changes in cholesterol levels. These data suggest that APOE4-driven exosomal pathway dysfunction could contribute to aging-dependent neuron vulnerability, cognitive impairment and AD risk. Endosomal material released into the extracellular space via exosomes is an important mechanism by which neurons remove debris [91], and failure to maintain an efficient exosome production and release in *APOEε4* carriers during ageing can disturb essential homeostatic and catabolic cellular processes, contributing to neuronal vulnerability and the risk of developing AD.

### 5.2. Amyotrophic Lateral Sclerosis (ALS) and Frontotemporal Dementia (FTD)

For many years believed to be two separate clinical entities and classified as pure movement and cognitive neurological disorders, ALS and FTD are now considered as a continuous, although complex, phenotypical spectrum [92]. The view rapidly changed when, besides the already discovered genes causing exclusively ALS—superoxide dismutase-1 (*SOD1*)—or FTD—progranulin (*GRN*) and microtubule-associated protein Tau (*MAPT*)—the GGGGCC hexarepeat expansion in chromosome 9 open reading frame 72 (*C9orf72*) gene was identified in 30%–50% of familial ALS, around 25% of familial FTD and about 5% of sporadic ALS and FTD, indicating that *C9orf72* is the major genetic factor in both conditions. This connection was further reinforced by the discovery of other genes mutated in both conditions, such as TAR DNA-binding protein-43 (*TDP-43*), fused in sarcoma (*FUS*) and sequestosome-1 (*SQSTM1*). Mutations in all these genes have toxic effects for the cell through two main convergent pathogenic mechanisms, aberrant RNA processing and protein degradation. In addition, more than 100 genetic loci predisposing to ALS/FTD have been identified by GWAS studies, although only a few of these genes have been validated by more than one study and, by consequence, their role in ALS/FTD remains uncertain [92].

Both wild-type and mutant proteins implicated in ALS/FTD have been detected in extracellular vesicles (comprising exosomes) derived from neurons and astrocytes, suggesting that they are a means of spreading the disease across the brain through a prion-like propagation mechanism of misfolded proteins [93]. These include SOD1, TDP-43, FUS and dipeptide repeat proteins (DPRs) derived from non-ATG translation (RAN-translation) of *C9orf72* hexanucleotide repeat expansion [94,95,96,97,98,99,100].

In 2017, Aoki and collaborators observed a lower number of multivesicular endosomes as well as a reduction in the number and secretion of exosomes in both ALS/FTD patient-derived fibroblasts and induced pluripotent stem cell-derived motor neurons carrying the *C9orf72* hexanucleotide repeat expansion [84]. They also demonstrate that C9orf72 directly interacts with the RAB7L1 GTPase to regulate trans-Golgi network vesicle trafficking in human neurons and fibroblasts. Moreover, increased expression of the autophagosome markers p62/sequestosome 1 and LC3-II was observed in C9ALS/FTD patient-derived fibroblasts and swollen autophagosomes in iPSC motor neurons, suggesting an impaired degradation of autophagosomes by lysosomal enzymes. Importantly, the phenotype of C9ALS/FTD fibroblasts and induced pluripotent stem cell neurons was recapitulated by *C9orf72* and *RAB7L1* knockdown in SH-SY5Y cells, as confirmed by a reduced amount of the exosome-specific markers ALIX and TSG101 and exosome surface antigens CD9, CD63 and CD81. In addition, silencing of *C9orf72* was able to reduce the expression of the autophagy marker p62. On the contrary, overexpression of both *C9orf72* and *RAB7L1* is capable to upregulate exosome secretion [84]. Interestingly, another study showed that exosome biogenesis and secretion is also impaired in C9ALS-derived astrocytes, and these defects are accompanied by a dysregulated miRNA cargo [85]. These data point to a fundamental role of C9orf72 in the regulation of exosome biogenesis, vesicle trafficking and also autophagy in neurons and astrocytes. This function seems to be mediated by its interaction with Rab GTPases, a family of proteins involved in endosomal trafficking, as described above [47,48,49,50,51]. Indeed, the C9orf72 protein contains a Differentially Expressed in Normal and Neoplasia (DENN) domain, which functions as GDP/GTP exchange factor (GEF) for the Rab GTPases. In support of this function, C9orf72 was also shown to serve as an effector of another GTPase, Rab1a, by interacting with it preferentially in its GTP-bound state, thereby controlling autophagy initiation. In keeping with this role, C9orf72 depletion in cell lines and primary neurons leads to the accumulation of p62-positive inclusions, and C9ALS/FTD patient-derived iPSC neurons show reduced levels of autophagy [101]. Ultimately, disrupted interaction of C9orf72 with Rab GTPases caused by the hexarepeat expansion mutation, resulting in compromised exosome formation, vesicle trafficking and autophagy, deserves attention as an emerging pathogenic mechanism in ALS/FTD.

FTD with parkinsonism related to chromosome 17 (FTDP-17) is an early-onset autosomal dominant form of FTD clinically characterized by parkinsonism, behavioral changes and dysfunction of personality [102,103] caused by mutations in either the *GRN* (FTDP-17U form) or *MAPT* gene (FTDP-17T form) [92,104,105,106]. Pallido-ponto-nigral degeneration (PPND) is a hereditary neurodegenerative syndrome belonging to the FTDP-17T group of disorders that is specifically caused by the c.837T>G (p.N279K) mutation in *MAPT*, which represents one of the most frequent mutations in FTDP-17T patients. This *MAPT* mutation induces alternative splicing of exon 10, resulting in a modification of the microtubule-binding region of Tau. In PPND/FTDP-17 patients iPSC-derived neural stem cells (NSCs), the N279K tau mutation was shown to induce an impairment of endocytic trafficking and accumulation of both endosomes and exosomes, as well as a reduction of lysosomes specific to the neuronal lineage [86]. Consistently, the levels of the intracellular/luminal vesicle and exosome marker flotillin-1 were significantly increased in the frontal and temporal cortex of PPND/FTDP-17 patients with the N279K tau mutation but remained unchanged in the occipital cortex, which is the most spared cortical region in the patients. These results suggest that alterations of intracellular vesicle trafficking and exosome secretion in neurons can constitute a relevant disease mechanism, underlying the neurodegenerative process in PPND/FTDP-17 patients with the N279K tau mutation.

Another recent work demonstrated that exosomes released by human primary fibroblasts contain progranulin mainly in its glycosylated form [87]. Moreover, by analyzing the fibroblasts of FTDP-17 patients carrying *GRN* null mutations and control subjects, they observed a strong reduction of progranulin in exosomes released by *GRN* mutant cells. Of note, the level of the exosomal marker TSG101 and the total protein cargo were significantly reduced in exosomes released by *GRN* mutant fibroblasts. In addition, nanoparticle tracking analysis showed a decrease in the total number of exosomes from *GRN* mutant human fibroblasts with respect to controls, as also confirmed by lower levels of CD63 positive vesicles detected by immunoassay with a specific antibody. Taken together, these data proved that null mutations in *GRN* strongly reduce the number of released exosomes and alter their composition.

### 5.3. Huntington’s Disease (HD)

HD is an autosomal dominant progressive neurodegenerative disorder caused by the expansion of a CAG trinucleotide repeat in exon 1 of huntingtin (*HTT*) gene, which generates a mutant huntingtin protein (mHtt) with an abnormally long polyglutamine tract at the N-terminus that confers toxic gains of function [107]. Exosomes have been shown to cargo mHtt both in vitro and in vivo, thus being responsible for the propagation of the mutant protein. Indeed, the cell-to-cell propagation of mHtt carrying 103 CAG repeats via exosomes has been demonstrated in fibroblasts and SH-SY5Y neuroblastoma cells. Furthermore, wild-type mice injected with human exosomes carrying mHtt triggered a Huntington disease-like pathology in the animals, this being the first report of human-to-mouse exosome-mediated propagation of toxic proteins [108].

A recent work by Hong and co-workers showed that the levels of exosome markers ALIX and flotillin-1 decreased in the isolated exosomes but not in the lysates of HD140Q knock-in (KI) mice derived astrocytes and striatum compared with wild-type mice, indicating that mHtt is able to impair exosome secretion in HD KI mice without affecting their biogenesis [88]. Interestingly, they also demonstrate that small N-terminal mHtt fragments can accumulate in the nuclei and form aggregates, causing decreased secretion of exosomes from cultured astrocytes. Furthermore, mHtt was shown to decrease the expression of β-crystallin, a small heat shock protein that is enriched in astrocytes and mediates exosome secretion [88]. β-crystallin plays a protective role in different neurodegenerative disorders: For example, β-crystallin can prevent amyloid fibril formation and reduce the toxicity of Aβ peptide [109]; it also inhibits the aggregation of α-syn fibrils in PD [110]. Importantly, overexpression of β-crystallin rescues defective exosome release from HD astrocytes and mHtt aggregates in the striatum of HD140Q KI mice. Altogether, these data show that mHtt impairs exosome secretion, suggesting a non-cell-autonomous neurotoxic action of mHtt. The authors propose that, since astrocyte-derived exosomes can carry a neuroprotective cargo such as heat shock proteins [111] contributing to neuronal survival, the reduced secretion of exosomes from HD KI astrocytes may provide less protection to neurons in the HD mouse brains.

### 5.4. Parkinson’s Disease (PD)

PD, the second most common neurodegenerative disease after AD, is a progressive movement disorder characterized by typical motor symptoms (bradykinesia, muscular rigidity, rest tremor, postural and gait impairment) that are caused by the degeneration of dopaminergic neurons in the substantia nigra. The major pathological hallmark of PD is the presence of Lewy bodies, which are mainly formed by fibrillary aggregation of misfolded α-syn [112].

Both dominant and recessive monogenic forms of PD are known. Six genes have been identified so far that cause autosomal dominant forms of PD (*SNCA*, *LRRK2*, *VPS35*, *EIF4G1*, *DNAJC13* and *CHCHD2*), while three genes (*Parkin*, *PINK1* and *DJ-1*) are associated with autosomal recessive forms of PD. *SNCA*, which encodes the protein α-syn, was the first gene to be associated with inherited PD, although *SNCA*-related PD is rare. Missense mutations in the *SNCA* gene or increased α-syn expression make α-syn more prone to aggregation. Mutations in *LRRK2* and *Parkin* are the most common causes of autosomal dominant and recessive PD, respectively. The greatest genetic risk factor for developing PD is the presence of a mutation in the *GBA* gene, encoding the lysosomal enzyme β-glucocerebrosidase, whose carriers have a fivefold increased risk of developing the disease. Additional genes associated with parkinsonism have been identified, namely *ATP13A2*, *C9ORF72*, *FBXO7*, *PLA2G6*, *POLG1*, *SCA2*, *SCA3*, *SYNJ1* and *RAB39B* [112].

Exosomes have been suggested to have a dual role in PD [113,114]. On the one hand, they can mediate the spread of toxic α-syn between cells more efficiently than free α-syn [115] and can accelerate the aggregation of exogenous α-syn [116]. The protein α-syn, for its part, can induce an increase of exosomal secretion by microglial cells leading to apoptosis of cortical neurons, suggesting that exosomes from activated microglia may be important mediators of α-syn induced neurodegeneration in PD [117]. Interestingly, CSF exosomes derived from PD patients contain α-syn pathogenic species and can induce the oligomerization of soluble α-syn in target cells [9], supporting the idea that exosomes may contain α-syn seeds that spread α-syn pathological aggregates in the brain. On the other hand, the reduction of α-syn intracellular levels by externalization via exosomes can have beneficial effects on neuronal survival [118,119], and PD derived microvesicles have been shown to exert a protective role in models of neuronal stress [120].

Some genes linked to PD have roles in endocytic pathways and autophagy, including *LRRK2*, *VPS35* and *PARK9*. *LRRK2* encodes the leucine-rich repeat kinase 2, a large multidomain protein released in association with exosomes involved in protein sorting and trafficking as well as autophagy [121,122]. Mutations in *LRRK2* account for about 4% and 1% of familial and sporadic PD, respectively [112]. Pathogenic *LRRK2* missense mutations have been shown to increase the proportion of protein that is autophosphorylated, particularly at the serine 1292 residue, a modification that is required for LRRK2 neurotoxicity. Interestingly, elevated levels of Ser(P)-1292 LRRK2 were found in both urinary and CSF exosomes derived from PD patients carrying an *LRRK2* mutation compared to controls, and the levels of LRRK2 autophosphorylation correlated with the severity of cognitive impairment [123,124]. VPS35 (vacuolar protein sorting 35) is involved in endosomal trafficking of proteins between the plasma membrane, Golgi apparatus, and lysosomes. The D620N mutation in *VPS35* has been linked to late-onset PD and leads to endosomal alterations and trafficking defects [125]. *PARK9* is also known as *ATP13A2* (ATPase cation transporting 13A2) and encodes a P-type transport ATPase found in MVBs. Loss-of-function mutations in *ATP13A2*/*PARK9* cause Kufor–Rakeb syndrome (KRS), a disorder characterized by juvenile-onset parkinsonism and cognitive decline [119]. Of note, overexpression of ATP13A2 suppresses a-syn toxicity in primary neurons [126]. Two published works suggest that mutations in *ATP13A2* affect exosome biogenesis and release. In their article, Kong and colleagues show that *ATP13A2* encodes a zinc pump and that neurospheres derived from a compound heterozygous *ATP13A2*-/- patient and ATP13A2 knockdown cells are 10-fold and 2-fold more sensitive to zinc, respectively; on the contrary, ATP13A2 overexpression confers zinc resistance in primary neurons. Moreover, they demonstrate that ATP13A2 localize to MVBs and that elevated ATP13A2 expression causes a 3-fold increase in the amount of α-syn associated with exosomes, while ATP13A2 knockdown decrease α-syn externalization via exosomes. Authors hypothesize that ATP13A2, by modulating the zinc levels in MVBs, can regulate the biogenesis of exosomes and propose a potential neuroprotective role of exosomes in PD [89]. Another study analyzed fibroblasts derived from KRS patients carrying a homozygous missense mutation or a compound heterozygous frameshift mutation in *ATP13A2*/*PARK9*, showing that loss of ATP13A2 function in mutant fibroblasts leads to a significantly lower number of intraluminal vesicles in MVBs and released exosomes compared with wild-type fibroblasts. By contrast, overexpression of ATP13A2 resulted in an increase of released exosomes in human H4 cells and mouse primary cortical neurons. Moreover, loss of ATP13A2 function led to decreased secretion of α-syn into extracellular space, whereas overexpressed ATP13A2 promotes the secretion of α-syn, at least in part via exosomes. Finally, ATP13A2 was found to regulate exosome biogenesis through functional interaction with the ESCRT pathway, as demonstrated by the evidence that ATP13A2 overexpression had no effect on exosome production in TSG101 silenced cells [90]. Taken together, these data suggest that ATP13A2/PARK9 has an important role in the biogenesis and release of exosomes, as well as in α-syn secretion, and raise the possibility that disruption of these pathways in patients with KRS contributes to the disease pathogenesis.

## 6. Concluding Remarks

Despite studies about the association between mutations in known ND genes and impairment of generation, composition and secretion of exosomes are still in their infancy; a picture is clearly emerging: The exosomes seem to play a dual role in the progression of NDs. If, on the one hand, they contribute to the spreading throughout the nervous system of toxic protein species boosting the neurodegenerative process, on the other hand, their cargo may exert a protective role by providing the neurons with proteins important for their survival as well as for cell-to-cell communication. A crucial point is that the identification of the above exosome functions is currently a challenge because the molecular features of both exosome types are now not well defined. The identification of a cargo-signature might allow defining a kind of clinical cargo-molecular fingerprint, leading to the identification of the affected releasing cell type. Moreover, new insights concerning exosome cargo and functions and the control of exosome biogenesis, including the association with genetic mutations, will allow improving the predictive diagnostic power of exosomes. Another important issue, even though it was not the focus of this review, is their possible recover in easily accessible body fluids. The determination of specific exosome molecular profiles will address the development of biomarkers and innovative therapeutic tools for ND diagnosis and treatment. Even if mechanisms of exome biogenesis as well as those of cargo recruitment are not so well defined, the promising perspectives in diagnosis and cure improvement might trigger more interest of scientists providing new insights in the field.

## Figures and Tables

**Table 1:** Biochemical composition of exosomes.

Donor Cells	Exosome Components
All types of cells	***Lipids:*** Cholesterol, phosphatidylserine (PS), sphingomyelins, saturated fatty acids, ceramide derivatives; ***Proteins:*** Membrane transport/fusion: Flotillins, RABs, annexins, GPI anchored proteins; adhesion molecules and transmembrane proteins: Tetraspanins (CD9, CD63, CD81 and CD82), integrins, LAMP; ESCRT components: ESCRT proteins, TSG101, ALIX, syndecan, syntenin; antigen presenting molecules: MHC class I and class II; cytoskeletal proteins: Actin, tubulin, profiling, cofilin; enzymes: GAPDH, PK, elongation factors; heat shock proteins and chaperones: HSP70, HSP90, HSC70; cytosolic proteins: Histones, ribosomal proteins, proteasome; ***RNA molecules:*** mRNAs, microRNAs, non-coding RNAs
Neurons	***Proteins:*** GluR2/3, AMPA receptors, L1CAM2, NCAM1 and NCAM2, Arc, MAP1B, Syt4, Evi/Wntless, Ephrins, APP, Amyloid-beta A4 precursor protein-binding proteins, NEDD4, cystatin C; ***RNA molecules:*** miR-125a, miR-124a, miR-132, let-7C, miR-21, miR-1973
Oligodendrocytes	***Proteins:*** GTPase Rab35, myelin proteolipid protein (PLP), myelin basic protein (MPB), myelin oligodendrocyte glycoprotein (MOG), 2′3′-cyclic-nucleotide-phospho diesterase (CNPase) stress-protective proteins, SOD, catalase, DM20; ***RNA molecules:*** miR-219
Microglia	***Lipids:*** Phosphatidylserine (PS); ***Proteins:*** Thrombospondin-1 and -4, TNF-α, IL-1B, IL-10, CCL2, P2X7R, GRIN2D, CD13; ***Other molecules:*** Serotonin (5HT), endocannabinoid; ***RNA molecules:*** miR-155, miR-146-5p
Astrocytes	***Proteins:*** Synapsin-1, FGF-2, VEGF, endostatin; ***RNA molecules:*** miR-29b

**Table 2:** Genetic alterations in ND genes affecting exosome biology.

Disease Name	Gene Name	Mutations	Effect on Exosomes	References
AD	*APOE*	ε4 allele	Decrease in exosome levels caused by the impairment of exosome biogenesis	[83]
ALS/FTD	*C9orf72*	Hexanucleotide repeat expansion	Reduction in the number and secretion of exosomes	[84,85]
	*MAPT*	N279K	Impairment of intracellular vesicle trafficking and exosome secretion	[86]
	*GRN*	Null mutations	Reduction in the number of released exosomes	[87]
HD	*HTT*	140Q expansion	Impaired exosome secretion	[88]
PD	*PARK9*/*ATP13A2*	T517I, frameshift mutations	Decreased number of the intraluminal vesicles in MVBs; diminished biogenesis and release of exosomes	[89,90]

AD = Alzheimer’s Disease; ALS = Amyotrophic Lateral Sclerosis; FTD = Frontotemporal Dementia; HD = Huntington’s Disease; PD = Parkinson’s Disease.
